# Supplementary figures and images for: Reassessing public opinion of captive cetacean attractions with a photo elicitation survey
Source: PeerJ. 2018 Nov 20;6:e5953. doi: 10.7717/peerj.5953 (PMC6251344; doi:10.7717/peerj.5953)

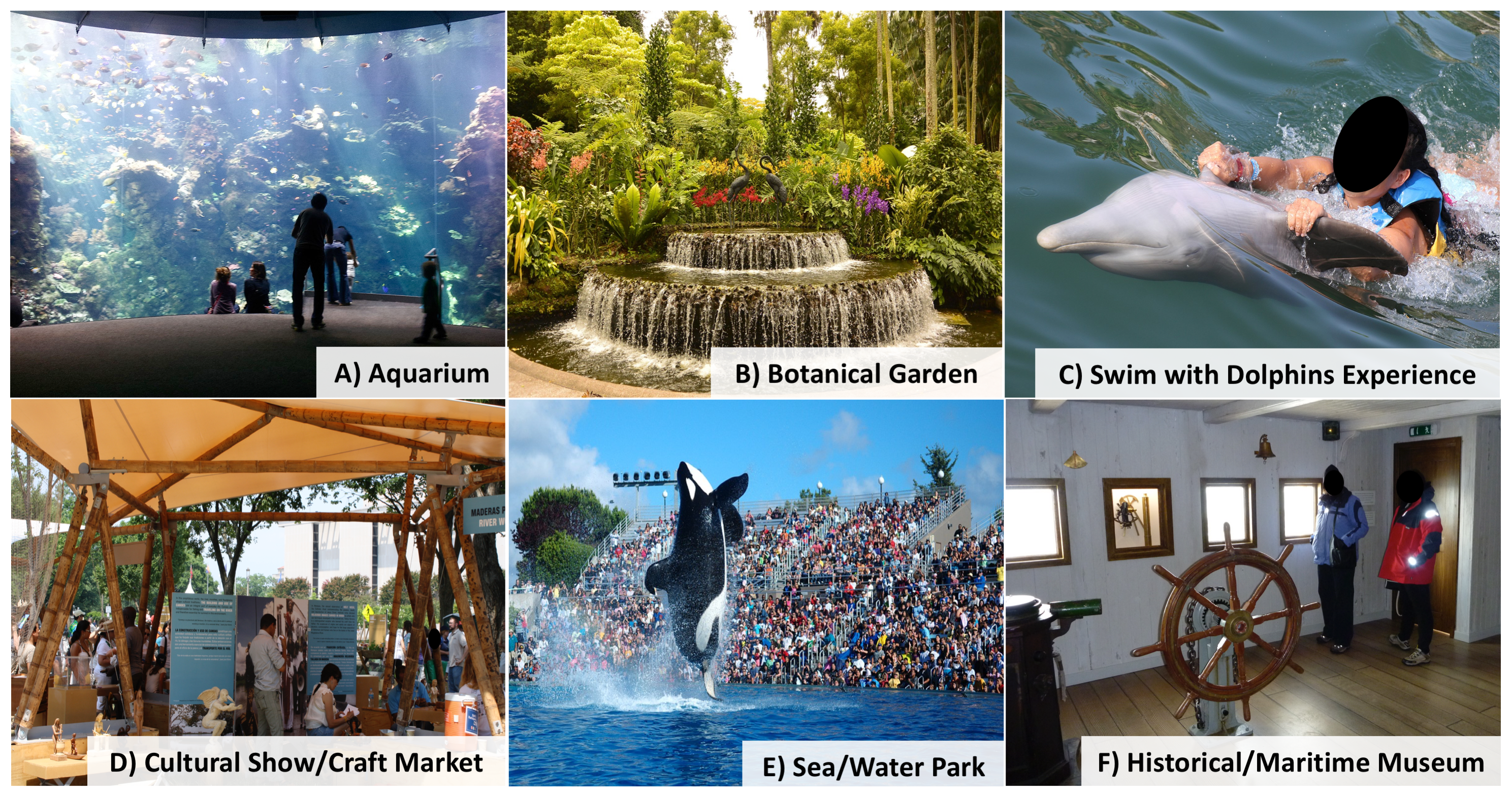

Supplement: Figure S1 — (A) Photograph of Aquarium, very similar to that shown to respondents; image credit: Maya Visanathan / Wikimedia Commons / CC-BY-SA-3.0 (B) Photograph of Botanical Gardens very similar to that shown to respondents; image credit: Max Pixel at https://www.maxpixel.net / CC0-1.0. (C) Photograph of SWTD attraction, very similar to that shown to respondents; image credit: Ohpencap / Wikimedia Commons / CC-BY-SA-4.0. (D) Photograph of Craft Market shown to respondents; image credit: Sophia Wassermann. (E) Photograph of MMP killer whale show, very similar to that shown to respondents; image credit: Yathin S Krishnappa / Wikimedia Commons / CC-BY-SA-4.0. (F) Photograph of Maritime Museum shown to respondents; image credit: Sophia Wassermann. [file peerj-06-5953-s001.png]
